# Iatrogenic air embolism

**DOI:** 10.1002/ccr3.3007

**Published:** 2020-06-10

**Authors:** Bryan Stringer, Lucie Henry, Raymond Foley

**Affiliations:** ^1^ Department of Internal Medicine University of Connecticut Farmington Connecticut USA; ^2^ Department of Pulmonology/Critical Care University of Connecticut Farmington Connecticut USA

**Keywords:** air embolism, biopsy, critical care, hyperbaric oxygen

## Abstract

Air embolism should be treated promptly with high fraction of supplemental oxygen and repositioning to help facilitate reabsorption of the air bubble. Hyperbaric oxygen therapy should be given to those with severe disease.

## Graphical abstract

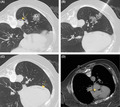

A 70‐year‐old woman with a past medical history of chronic obstructive pulmonary disease presented to the hospital for computed tomography (CT)–guided biopsy of a new left lung nodular opacity (Figure 1) (Panel B). During the procedure, she developed dyspnea, dizziness, and a sinus bradycardia. It was noted on CT imaging that there was air in the descending aorta (Panel A, arrow), left ventricle (Panel C, arrow), and left ventricular outflow tract (Panel D, arrow). The procedure was aborted, and the patient was placed in a left lateral decubitus position with Trendelenburg and supplemented with 100% FiO2 by non‐rebreather mask. She was transferred to the ICU for further monitoring. All symptoms were resolved, and the heart rate was normalized. Hyperbaric oxygen therapy (HBOT) was considered; however, it was deferred since her symptoms were resolved and she was rapidly titrated down to nasal cannula. A repeat CT scan of the chest and head and echocardiogram were performed with no further air visualized. Patients with air embolism should be treated promptly with high fraction of supplemental oxygen and repositioning to help facilitate reabsorption of the air bubble.^1^ HBOT should be administered to those with end‐organ damage, neurological deficits, or evidence of cardiopulmonary compromise.^2^

**Figure 1:**
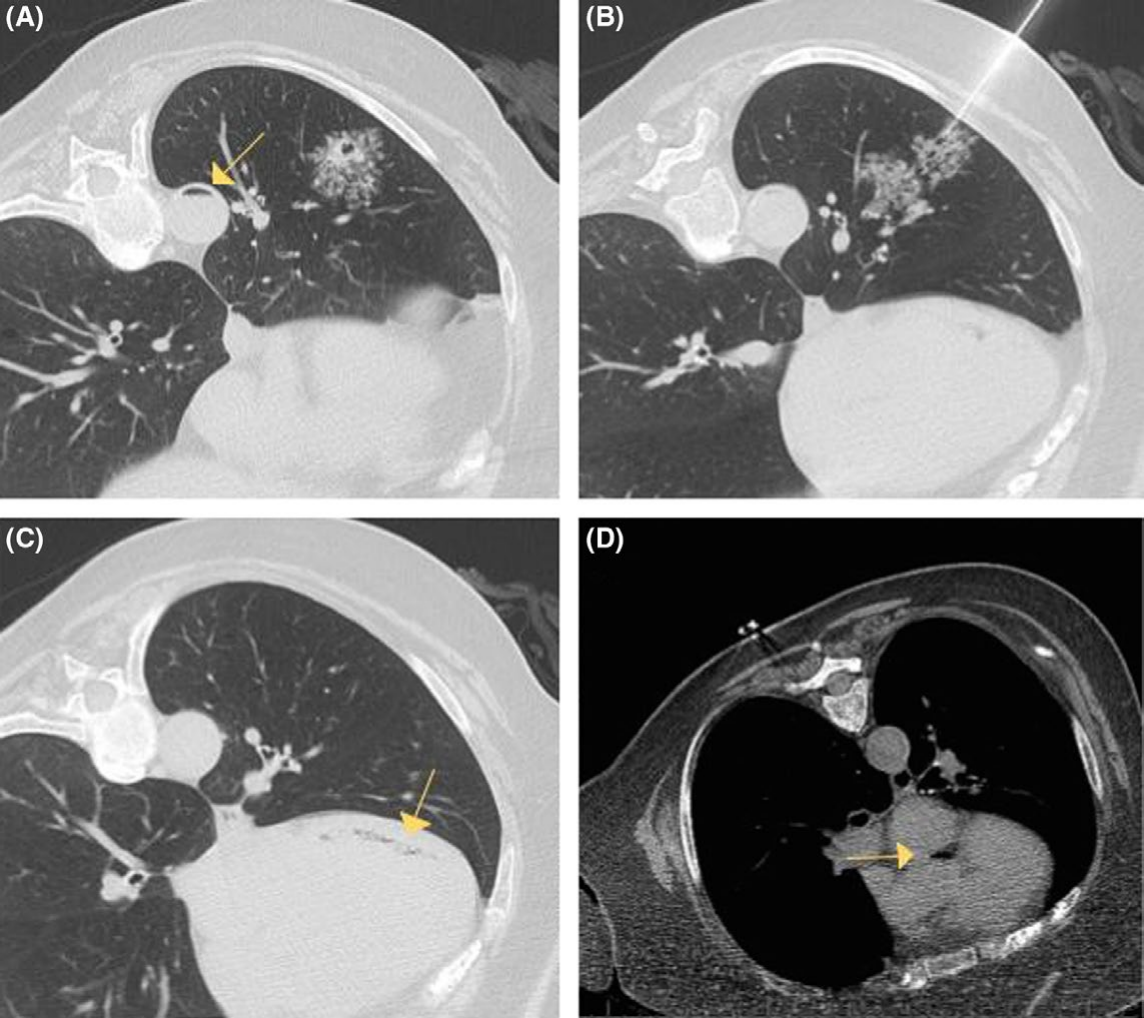
Computed tomography (CT) during biopsy demonstrating multiple areas of intravascular air

## CONFLICT OF INTEREST

There are no conflicts of interest.

## AUTHOR CONTRIBUTIONS

BS: was responsible for conception and design, writing the case description, and editing of the computed tomography images. LH: assisted in writing case description and participated in literature review to construct discussion points in manuscript. RF: supervised conception and design, and revised manuscript and images in entirety.

## INFORMED CONSENT

Informed patient consent was obtained for publication of the case details.
